# Successful triple therapy for advanced synchronous multiple primary esophageal carcinoma with metal stenting, photodynamic and comprehensive systemic therapies—shining light on hope: a case report and literature review

**DOI:** 10.3389/fimmu.2025.1580616

**Published:** 2025-04-01

**Authors:** Dan Zou, Ying Yan, Yifan Li, Huanhuan Ma, Yuping Bai, Xueyan Wang, Bofang Wang, Yunpeng Wang, Jingwei Ma, Hao Chen

**Affiliations:** ^1^ The Second Hospital and Clinical Medical School, Lanzhou University, Lanzhou, China; ^2^ Department of Magnetic Resonance Imaging, The Second Hospital and Clinical Medical School, Lanzhou University, Lanzhou, China; ^3^ Gansu Provincial Key Laboratory of Environmental Oncology, The Second Hospital and Clinical Medical School, Lanzhou University, Lanzhou, China; ^4^ Department of Surgical Oncology, The Second Hospital and Clinical Medical School, Lanzhou University, Lanzhou, China; ^5^ The Key Laboratory of Humanized Animal Models, The Second Hospital and Clinical Medical School, Lanzhou University, Lanzhou, China

**Keywords:** synchronous multiple primary esophageal cancer, photodynamic therapy, metal stent implantation, chemotherapy, targeted therapy, immunotherapy

## Abstract

Synchronous multiple primary esophageal cancer (SMPEC) is a rare and aggressive condition often accompanied by obstructive dysphagia, significantly impacting patients’ quality of life. Current treatments, including chemotherapy, radiotherapy, immunotherapy, and targeted therapy, are limited in providing immediate symptom relief. This case report describes a 64-year-old female with SMPEC and metastases to thoracic lymph nodes, the lesser curvature of the stomach, and the right adrenal gland, presenting with severe dysphagia (score 4 on the Japanese Dysphagia Severity Scale). To rapidly alleviate symptoms, she underwent simultaneous metal stent implantation and photodynamic therapy (PDT). She started a liquid diet on the second day after treatment and resumed a normal diet one week later. Subsequently, she underwent systemic chemotherapy, targeted therapy, and immunotherapy. By the third treatment cycle, primary and metastatic lesions significantly decreased, achieving a partial response (PR) with stable disease and progression-free survival (PFS) exceeded 12 months. This triple therapy approach—combining stent implantation, PDT, and systemic treatments—proved effective and safe for advanced SMPEC, not only providing immediate dysphagia relief and selective tumor destruction but also delaying disease progression and improving patient outcomes.

## 1 Introduction

Esophageal cancer is a common malignant tumor of the digestive tract. According to the latest statistics from 2024, there were over 510,000 new cases globally in 2022, resulting in approximately 445,000 deaths (1). In China, the number of new cases reached 224,000 in 2022, accounting for a significant proportion of global cases (2). Early esophageal cancer often has no obvious symptoms. As a result, most patients are diagnosed at an advanced stage, leading to a poor prognosis and a five-year survival rate of less than 5% (3). Synchronous multiple primary esophageal cancer(SMPEC) is a rare and aggressive malignancy. It is characterized by the presence of two or more distinct cancerous lesions in different areas of the esophagus. These lesions may appear simultaneously or within a six-month time frame (4). The incidence of SMPEC ranges from 0.1% to 10.0% and treatments for SMPEC commonly include surgical resection, radiotherapy, and chemotherapy (5). However, there are no standardized guidelines that systematically elaborate on SMPEC. A retrospective study indicates that patients with SMPEC accompanied by distant metastases have a median survival time of only 8.3 months (6). Several studies have revealed that SMPEC is often an independent factor for poor prognosis, with the prognosis of such patients being even worse (4, 7, 8). For patients with this condition, more advanced multidisciplinary treatment regimens should be considered.

Photodynamic therapy (PDT) is a highly selective and minimally invasive treatment modality. It utilizes specific photosensitizers (PS) that selectively accumulate in abnormal and rapidly proliferating tissues. It operates by destroying the target tissue through the interaction of light sources and oxygen. Compared with traditional treatment methods, PDT has advantages such as minimal trauma, low toxicity, high selectivity, a wide range of applications, and a low likelihood of developing drug resistance. However, PDT also has certain limitations, such as phototoxicity, the risk of perforation, and restricted penetration depth, which may limit its effectiveness when used alone, particularly in deeper or more advanced tumors. Given these challenges, a combination therapy approach is often required to enhance the efficacy of PDT, expand its therapeutic scope, and achieve better disease control. This report describes an SMPEC that underwent PDT simultaneously with metal stent implantation, which killed the tumor and relieved the patient’s dysphagia. After receiving systemic therapy, including chemotherapy, targeted therapy, and immunotherapy, the patient’s dysphagia completely disappeared. The primary and metastatic lesions were assessed as having achieved partial response (PR) in the short term, with long-term disease stability. The patient maintained a good quality of life without experiencing intolerable adverse effects.

## 2 Case report

### 2.1 Brief medical history and preliminary diagnosis

**Figure 1:**
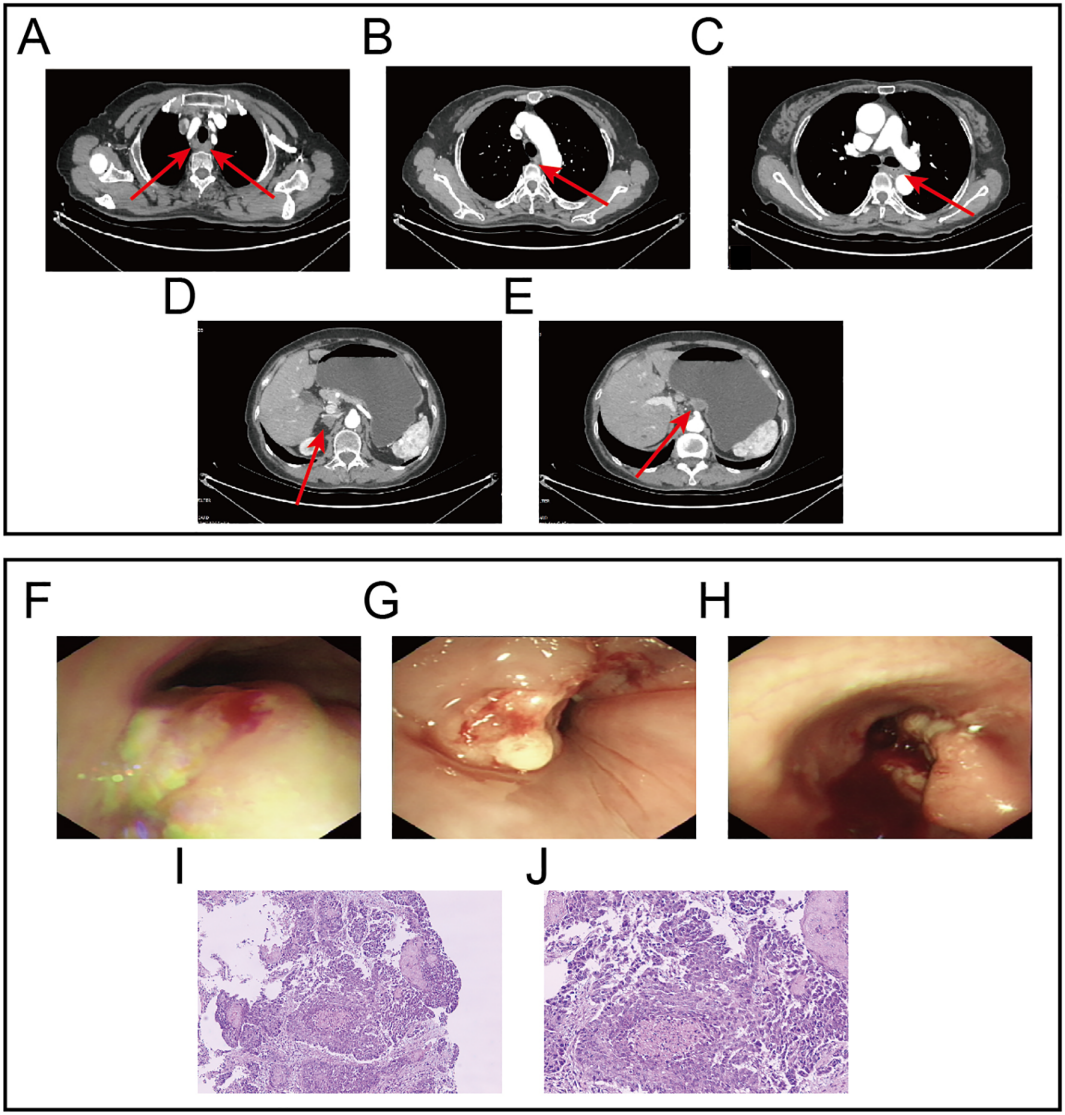
Gastroscopy images, Contrast-enhanced CT images and HE staining images at initial diagnosis. **(A)** Lesion in the upper segment of the esophagus and metastatic lymph nodes. **(B)** Lesion in the middle segment of the esophagus. **(C)** Lesion in the lower segment of the esophagus. **(D)** Metastatic lesion in the right adrenal gland. **(E)** Metastatic lesion on the lesser curvature side of the stomach.**(F)** Endoscopic findings revealed the first lesion located approximately 17-19 centimeters from the incisors. **(G)** Endoscopic findings revealed the second lesion located approximately 26-32 cm from the incisors. **(H)** Endoscopic findings revealed the third lesion located approximately 34-37 cm from the incisors. **(I, J)** Represent the HE staining results at 10x and 20x magnification, respectively.

A 64-year-old female patient was admitted with the chief complaint of “difficulty in swallowing for one month”. One month before admission, she developed dysphagia without any obvious triggering factors, being able to ingest only small amounts of liquid food. She was accompanied by acid reflux but without nausea, vomiting, abdominal pain or bloating. She had not received any treatment at other hospitals. There was no family history of cancer, and the physical examination revealed no positive findings. Contrast-enhanced computed tomography (CT) scan showed uneven thickening of the esophageal wall, approximately 8 cm in length, with a maximal thickness of about 0.9 cm. Enlarged lymph nodes were observed around the lesion, the largest of which measured approximately 16×14 mm, highly suggestive of esophageal cancer with lymph node metastasis. A round nodular shadow measuring approximately 17×11 mm was observed on the lesser curvature side of the stomach, highly suggestive of a metastatic tumor. A low-density nodular shadow measuring 15 × 10 mm was detected in the right adrenal area, also highly suggestive of a metastatic tumor (**Figures 1A–E**). Gastroscopy revealed multiple large, irregular, raised lesions separately located approximately 17-19 cm, 26–32 cm, and 34-37 cm from the incisors. Biopsies were taken at each lesion. Lesions in the middle and lower esophagus occupied about three-quarters of the lumen, suggesting advanced esophageal cancer (**Figures 1F–H**). Histopathological examination indicated squamous cell carcinoma (**Figures 1I, J**). Hematological analysis revealed a hemoglobin level of 85 g/L. Liver function tests showed an albumin level of 29 g/L. Electrolyte panel indicated a potassium level of 3.2 mmol/L. Renal function tests, coagulation profile, tumor markers, and electrocardiogram demonstrated no significant abnormalities.

Preliminary Diagnosis: The patient was diagnosed with synchronous multiple primary esophageal squamous cell carcinomas (cT3N2M1, Stage IVB); with local lymph node metastasis, a remote metastatic lesion on the lesser curvature side of the stomach, as well as metastasis in the right adrenal gland. Additionally, the patient was suffering from moderate anemia. The patient’s dysphagia score was 4, indicative of severe swallowing difficulty. Her ECOG performance status was 2, reflecting a moderate level of ambulatory and self-care ability, and her Karnofsky Performance Status (KPS) score was 60, suggesting a moderate level of functional impairment.

### 2.2 Therapeutic process

We conducted a multidisciplinary discussion for this SMPEC patient. To promptly alleviate dysphagia, our team performed endoscopic placement of metal stents and administered PDT. Systemic treatments including chemotherapy, targeted therapy, and immunotherapy were initiated to control disease progression. This comprehensive approach effectively relieved the patient’s symptoms and improved her quality of life.

#### 2.2.1 Endoscopic metal stent implantation

**Figure 2:**
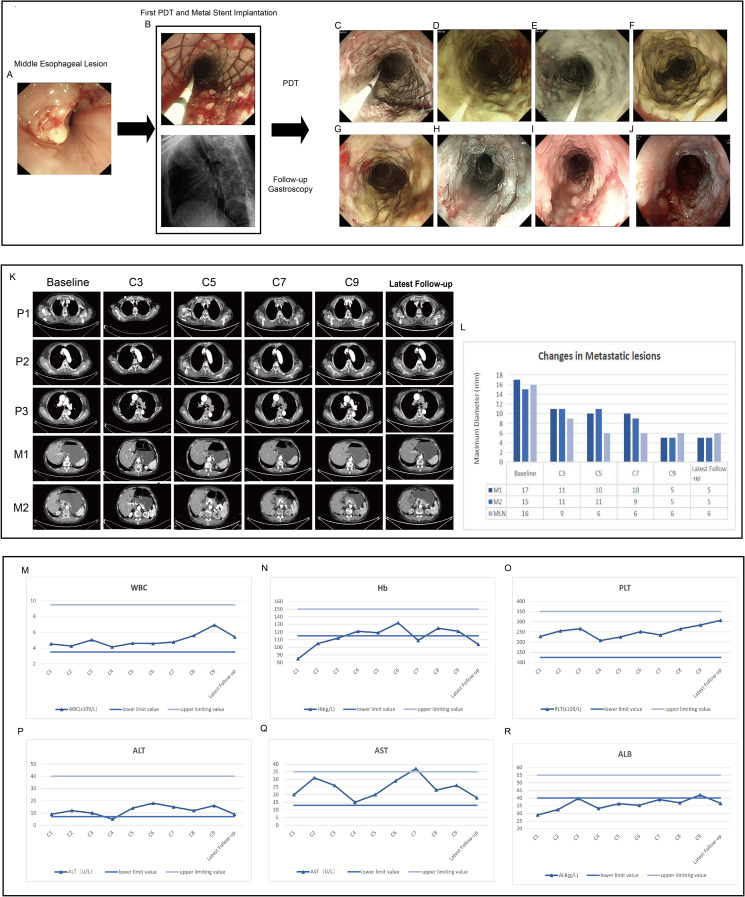
Endoscopic images, contrast-enhanced CT images and critical laboratory parameters before and after treatment. **(A)** Middle esophageal lesion prior to treatment. **(B)** Metal stent implantation and the first PDT to relieve obstruction at the middle segment lesion, intraoperative X-ray shows the stent is elongated in the middle, indicating that the stent is in place. **(C–E)** The second, third, and fourth PDT for the middle segment lesion of the esophageal cancer, respectively, and the mucosal tissue of the tumor gradually turns white and necrotic. **(F–J)** After one week, two months, five months, nine months and twelve months of treatment, lesion mucosa appeared white, necrotic, and detached, while partial esophageal mucosal roughness is observed. Post-treatment, the lumen remains patent. **(K, L)** Contrast-enhanced computed tomography demonstrated significant reduction in the size of the primary esophageal lesion and metastatic lesions after treatment. P1-P3 represent the primary lesions in the upper, middle, and lower segments of the esophagus, respectively. M1 indicates the metastatic lesion on the lesser curvature side of the stomach, and M2 indicates the metastatic lesion in the right adrenal gland. C3-C9 represent the re-examination every two cycles. MLN indicates the metastatic lymph nodes. **(M–O)** The changes in WBC, Hb, and PLT in the complete blood count during each treatment cycle. **(P–R)** The changes in ALT, AST, and ALB during each treatment cycle.

Under intravenous anesthesia, an endoscopic procedure was carried out in accordance with the tumor emergency treatment. Due to severe esophageal stenosis, the endoscope was unable to advance through the narrowed segment, so a yellow zebra guidewire was inserted under endoscopic guidance into the stomach cavity under X-ray visualization. Following the guidewire, an intestinal metal stent (22×120 mm, WallFlex, Boston Scientific) was deployed into the middle and lower segments of the esophageal lesions to alleviate dysphagia. After the successful placement of the metal stent, an X-ray scan was conducted to confirm that the stent was properly expanded along the esophageal axis with normal positioning. It was ensured that both sides of the stent were about 1.5 cm above the upper and lower boundaries of the lesion. The upper and lower edges of the stent showed no significant compression or distortion (**Figures 2A, B**).

#### 2.2.2 PDT

Forty-eight hours prior to the endoscopic procedure, the patient was transferred to the PDT ward and received an intravenous injection of Hematoporphyrin (Hiporfin, MaiLang Bio-Pharmaceutical Co., Ltd., China) at a dosage of 3 mg/kg. A cylindrical optical fiber (6 cm in length and 480 μm in diameter, SMA905 interface optical fiber system) and a semiconductor laser PDT device (PDT630II type, Guilin Xingda Photophysiotherapy Equipment Co., Ltd.) were used to introduce the fiber to the esophageal lesion site for the first PDT. Based on our team’s treatment experience and the energy calculation formula, which is as follows: E =(P_density × V) × t, where E represents the total energy (J), P_density represents the power density (W/cm^3^), V represents the tumor volume (cm³) and t represents the time (s). If the treatment is surface-based, the surface area rather than the volume, should be used. The lower segment was irradiated for 8 minutes at a power of 800 mW with an energy of 384 J, followed by the middle segment, which was irradiated for 10 minutes at a power of 800 mW with an energy of 480 J, and finally the upper segment, which was irradiated for 5 minutes at a power of 800 mW with an energy of 240 J. Based on our team’s treatment experience, we performed the second PDT the next day, with the same energy conditions as the first treatment. On the third and fourth days, we performed the third and fourth PDT, respectively, and adjusted the energy conditions to: each segment (lower, middle, and upper) was treated for 5 minutes with 800 mW power and 240 J energy (**Table 1**). During this process, the patient experienced no obvious discomfort, and the patient needed to avoid light for four weeks after PDT.

**Table 1:** Irradiation parameters for PDT in treating lesions at different locations.

	The upper segment	The middle segment	The lower segment
Lesion Length(cm)	2	6	3
Lesion Thickness(mm)	6	9	8
Irradiation Power(mW)	800	800	800
Irradiation time (min)			
Day1	5	10	8
Day2	5	10	8
Day3	5	5	5
Day4	5	5	5
Energy(J)			
Day1	240	480	384
Day2	240	480	384
Day3	240	240	240
Day4	240	240	240

#### 2.2.3 Systemic treatment

In addition to local treatments, systemic therapy is required to control the disease. The TX regimen is an effective chemotherapy protocol for esophageal cancer, widely used in late-stage patients who cannot undergo surgery. One week after the first PDT, the patient was administered the TX chemotherapy regimen: nab-paclitaxel (150 mg/m^2^, intravenous infusion on days 1 and 8); capecitabine (1000 mg/m^2^, taken orally from days 1 to 14); anlotinib (12 mg, taken orally from days 1 to 14), Tislelizumab (200 mg, intravenous infusion on day 1) constituting a 3-week cycle. Chemotherapy was continued until the completion of the 9 cycle, followed by maintenance targeted therapy and immunotherapy.

### 2.3 Therapeutic effect

#### 2.3.1 Endoscopic evaluation of therapeutic efficacy

During the initial PDT session, a metal stent was implanted, and the patient underwent PDT for four consecutive days. Endoscopic examinations confirmed stent stability and showed progressive mucosal necrosis and sloughing(**Figures 2A–E**). One week post-PDT, significant necrotic tissue was observed (**Figure 2F**). Follow-ups at one, four, nine, and twelve months post-PDT showed tumor size reduction, new mucosal growth, and sustained esophageal patency (**Figures 2G–J**).

#### 2.3.2 Evaluation of dysphagia

After completing the first treatment cycle, the patient’s dysphagia score showed a significant improvement, increasing by more than six points compared to the baseline. This score has remained at ≥9 since then, indicating a substantial alleviation of dysphagia symptoms.

#### 2.3.3 Evaluation of CT

The latest contrast-enhanced CT scan shows a significant reduction in the primary esophageal lesion. The paratracheal metastatic lymph node diameter decreased from 16 mm to 6 mm, the lesser curvature gastric metastasis from 17 mm to 5 mm, and the right adrenal metastasis from 15 mm to 5 mm (**Figures 2K, L**). According to iRECIST guidelines, the patient achieved a partial response (PR) for both primary and metastatic lesions.

#### 2.3.4 Evaluation of adverse reactions

Throughout the entire treatment course, the patient experienced only a mild decrease in albumin levels and moderate anemia. No intolerable bone marrow suppression, hepatic or renal dysfunction, hypertension, bleeding, or other common side effects associated with chemotherapy and targeted therapy were observed. No significant immune-related adverse effects were noted (**Figures 2M–R**). Additionally, no photosensitivity reactions, significant pain, bleeding, perforation, or infection were observed.

#### 2.3.5 Follow up

**Figure 3:**
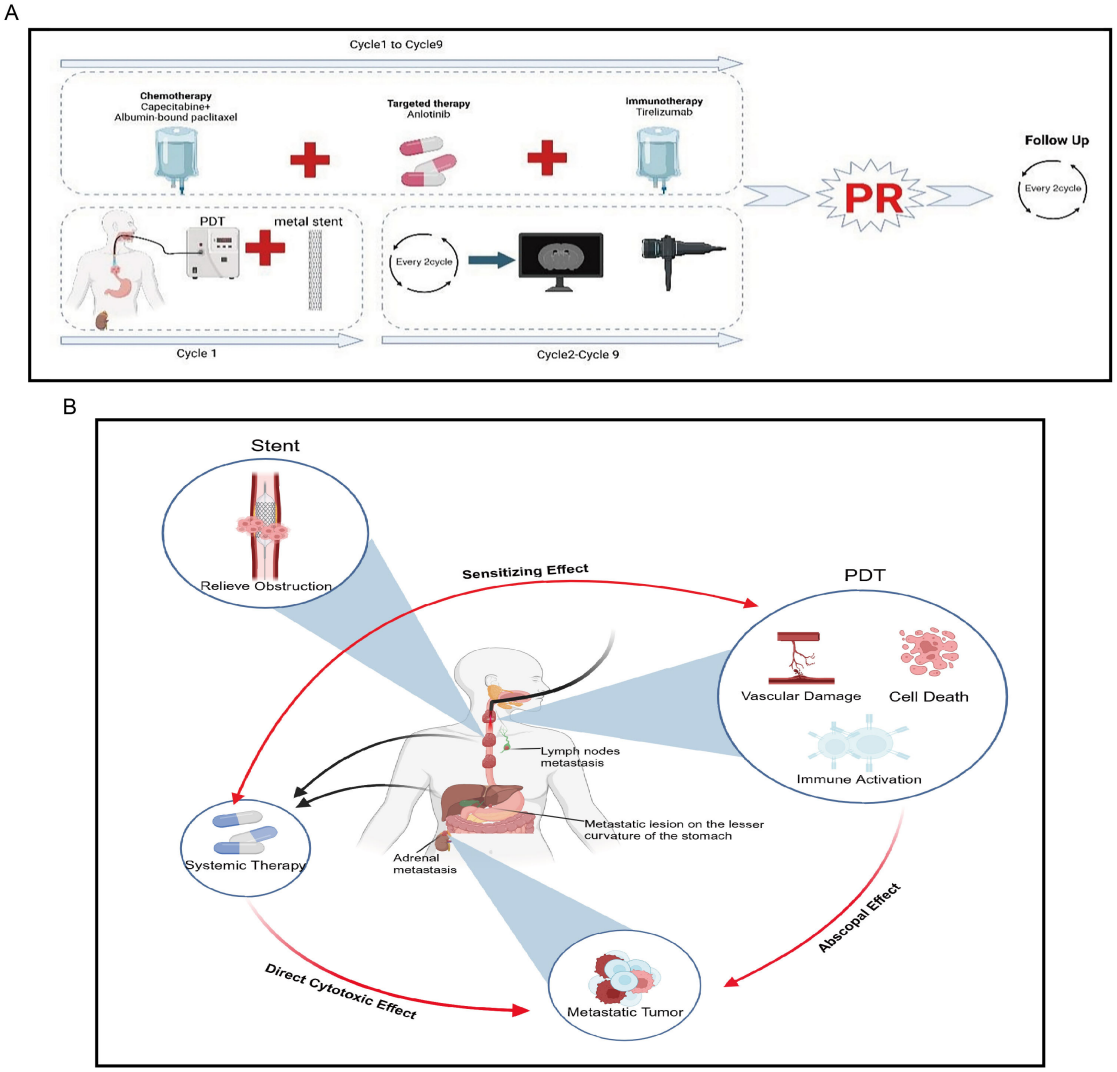
Overview of the patient’s treatment course reported in this study and brief mechanism of anti-tumor. **(A)** The treatment course. **(B)** Metal stents rapidly alleviate obstruction, PDT can exert local tumor-killing effects while generating “abscopal effects” and enhancing the therapeutic efficacy of systemic treatment.

Currently, the patient remains under regular follow-up and continues to receive treatment and monitoring at our hospital. As of the latest follow-up (12 months post-treatment), the patient remains in stable condition with no evidence of disease progression, indicating a progression-free survival (PFS) of at least 12 months. No late complications, including perforation, severe stenosis, or long-term phototoxicity, have been observed. No late complications, such as perforation or severe stenosis, have been observed. The treatment course and follow-up timeline are illustrated in **Figure 3A**. Moreover, the patient remains alive, and overall survival (OS) has not yet been reached (NR), with ongoing follow-up being conducted to assess long-term outcomes.

## 3 Discussion

The management of advanced esophageal cancer mainly involves palliative and multidisciplinary treatment strategies, with the primary aim of prolonging overall survival (OS), alleviating symptoms, and enhancing quality of life. Evidence from clinical studies highlights the limited efficacy of chemotherapy alone, with a reported median OS of only 9.1 months in patients with advanced esophageal squamous cell carcinoma (ESCC) (9). Doublet chemotherapy regimens based on 5-fluorouracil and platinum agents are recommended as first-line treatment options in various guidelines. Literature reports indicate that cisplatin combined with 5-FU as first-line chemotherapy has a response rate of approximately 30%, with a median OS of only 6.6 to 10.4 months (10–12). Recent advancements in targeted therapies and immunotherapies have significantly improved survival outcomes and quality of life in this patient population. A study suggests that apatinib monotherapy in patients with advanced esophageal cancer achieved a median PFS of 4.63 months and a median OS of 6.57 months (13). Multiple clinical studies have demonstrated that chemotherapy combined with immunotherapy significantly improves the prognosis of patients with advanced esophageal cancer (14–17). However, a considerable proportion of patients with advanced esophageal cancer still fail to benefit from immunotherapy. Although metal stent implantation is a key palliative treatment for relieving dysphagia and improving the quality of life in patients with advanced obstructive esophageal cancer, its use alone does not improve overall survival (18–20).

PDT has been applied alone or in combination with various solid tumors, showing significant efficacy, particularly in gastrointestinal tumors. Zeng R et al., in a retrospective study analyzing 32 patients with advanced obstructive esophageal cancer who underwent PDT, reported an effective rate of 78.1%, significantly relieving patients’ dysphagia symptoms (21). Yamashita et al. found that among 82 patients who received a first PDT, 27 underwent a second PDT. The local complete response (L-CR) rates for the first and second PDT were 63.0% and 40.7%, respectively. Therefore, a second PDT session proved to be an effective treatment method for local failure following the first PDT (21). A retrospective study involving 31 patients with esophageal cancer analyzed the outcomes after PDT. The OS for patients achieving complete response (CR) was 31.9 months, while those with PR had an OS of 28.2 months. The disease-free survival (DFS) for CR patients was reported to be 21.9 months. These findings suggest that PDT is a reasonable palliative treatment option for esophageal cancer (21). Our team previously reported a case of advanced recurrent esophageal cancer with dysphagia. This case highlights the potential of PDT as part of a combined therapeutic strategy, offering a novel approach for the management of advanced esophageal cancer (22). PDT is based on the interaction between PS, light of specific wavelengths, and oxygen, which generates reactive oxygen species (ROS) to destroy tumor or lesion tissues. In solid tumors, cancer cells have an increased uptake of LDL lipoproteins, and PS molecules have a high affinity for LDL, resulting in significantly higher accumulation of PS in cancer cells compared to normal cell (23, 24). The antitumor mechanisms of PDT mainly include three aspects:

1. Direct Killing of Tumor Cells: Multiple studies have found that PDT can induce tumor cell death, including apoptosis, necrosis, and autophagy (25–27).
2. Damage to Tumor Blood Vessels: Maas AL et al. found that the PS photofrin has an affinity for the collagen-containing vascular basement membrane, leading to tumor vascular damage in lung cancer mouse models after PDT (28). Multiple *in vivo* experiments have shown that PDT can cause damage to tumor vascular endothelium, slow down blood flow within tumor vessels, induce thrombosis, or significantly increase tumor vascular permeability, resulting in tumor vascular damage and ischemia (29).
3. Activation of Immune Responses Post-PDT: PDT induces immunogenic cell death in tumor cells, releasing tumor-associated antigens and activating the body’s antitumor immune responses (30, 31). Therefore, PDT not only directly kills tumor cells and destroys tumor blood vessels but also activates the body’s antitumor immunity, exerting comprehensive antitumor effects.

This represents the first report of applying PDT-based triple therapy to a rare case of an advanced SMPEC patient presenting with severe dysphagia. First, when combined with metal stents, PDT can rapidly alleviate dysphagia in patients, effectively eradicate tumors, and compensate for the limitations of other local therapies, such as the severe side effects of radiotherapy. Additionally, when used in combination with systemic therapy, PDT can enhance the therapeutic efficacy of both approaches, achieving an effect greater than the sum of its parts (1 + 1 + 1 + 1 + 1 > 5). Second, PDT can enhance the efficacy of systemic treatments. For example, PDT can damage tumor cell membranes, increasing membrane permeability, which enables chemotherapeutic drugs to enter the cells more easily and raises the intracellular concentration of the drugs (32). Studies have discovered that PDT synergizes with irinotecan to improve therapeutic outcomes in pancreatic cancer (33). PDT can also convert “cold tumors” into “hot tumors,” enhancing the efficacy of immune checkpoint inhibitors (34, 35). Our previous study demonstrated that PDT can reshape the tumor immune microenvironment in gastric cancer, leading to increased immune cell infiltration, enhanced immunotherapy efficacy, and prolonged survival in patients with advanced disease (34). Similarly, Tong Q et al. reported that combining Pheophorbide A-mediated PDT with αPD-L1 therapy upregulated intratumoral PD-L1 expression and promoted T cell infiltration, further enhancing the effectiveness of immunotherapy (36). Additionally, while exerting local anti-tumor effects, PDT can also kill metastatic lesions, producing an “abscopal effect”. PDT induces neutrophil infiltration into the tumor and activates tumor-specific cytotoxic CD8^+^ T cells, thereby stimulating anti-tumor immunity and killing tumor cells. The activity of these activated CD8^+^ T cells is not confined to the local tumor area but may also target distant sites such as metastases (37). A study using a colorectal cancer BALB/c mouse model found that PDT can recruit lymphocytes and inhibit the growth of distant, unirradiated metastatic lesions (38). Other studies have found that PDT induces the release of several immunostimulatory molecules, namely damage-associated molecular patterns (DAMPs), such as ATP, calreticulin, high-mobility group box 1 protein (HMGB1), heat shock proteins 70 and 90 (HSP 70 and 90), and cytokines/chemokines, thereby enhancing innate and adaptive immunity (39). Therefore, PDT not only directly kills tumor cells but also produces an “abscopal effect”, synergizing with chemotherapy, targeted therapy, and immunotherapy to enhance anti-tumor efficacy (**Figure 3B**).

Based on this case report, treating patients with SMPEC is highly challenging, and traditional monotherapies often fail to yield satisfactory outcomes. The comprehensive application of PDT combined with metal stent implantation, chemotherapy, targeted therapy, and immunotherapy provides an integrated approach of local control and systemic treatment for these patients, improving survival rates and quality of life. Nevertheless, this is a single-case report with limited generalizability. Moreover, the interactions between different drugs may involve multiple mechanisms, making combination therapy highly complex. Our team is currently conducting a single-center clinical trial to validate the efficacy, safety, and clinical applicability of this combination therapy (ChiCTR2200064280, ChiCTR2300076208). In the future, we aim to conduct large-scale, multicenter clinical trials to further confirm these findings.

## Data Availability

The original contributions presented in the study are included in the article/supplementary material. Further inquiries can be directed to the corresponding author.
